# Encoding Genes of Metallo β‐Lactamases (*IMP*, *NDM*, and *VIM*) in *Klebsiella Pneumoniae* in Iran: A Systematic Review and Meta‐Analysis

**DOI:** 10.1002/hsr2.70927

**Published:** 2025-06-18

**Authors:** Hamid Sadeghi, Susan Khanjani, Masoumeh Aslanimehr, Nima Aria, Mohammad Reza Maleki, Farhad Nikkhahi, Nematollah Gheibi, Seyed Mahmoud Amin Marashi, Saeideh Gholamzadeh khoei

**Affiliations:** ^1^ Medical Microbiology Research Center Qazvin University of Medical Sciences Qazvin Iran; ^2^ Student Research Committee Qazvin University of Medical Sciences Qazvin Iran; ^3^ Clinical Research Development Unit, Kowsar Hospital Qazvin University of Medical Sciences Qazvin Iran; ^4^ Cellular and Molecular Research Center, Research Institute for Prevention of Non‐Communicable Diseases Qazvin University of Medical Sciences Qazvin Iran; ^5^ Social Determinants of Health Research Center, Research Institute for Prevention of Non‐Communicable Diseases Qazvin University of Medical Sciences Qazvin Iran

**Keywords:** *Klebsiella pneumonia*, metallo‐β‐lactamases, microbial drug resistance

## Abstract

**Background and Aim:**

Antimicrobial resistance is one of the top 10 global public health threats, particularly growing resistance to β‐lactam antibiotics, including carbapenems, which are among the last‐resort antibiotics used against multidrug and extensively drug‐resistant bacteria.

**Methods:**

This meta‐analysis explores the status of class B carbapenemases, including Imipenemase (*IMP*), New Delhi metallo‐β‐lactamase (*NDM*), and Verona integron‐encoded metallo‐β‐lactamase (*VIM*) in *Klebsiella pneumoniae* in Iran. Eligible articles were retrieved from electronic literature searches of online databases (PubMed, Scopus, Wiley Online Library, ScienceDirect, and Google Scholar) to identify studies published between 2010 and 2024.

**Results:**

The study selection process yielded 2517 studies. A total of 124 studies fulfilled the inclusion criteria; of these, 25 studies were eligible for meta‐analysis. A meta‐analysis of prevalence studies demonstrated that the most encoding genes of MBLs were to NDM, VIM, and IMP at 7.1% (95% confidence interval [CI]: 4.9%–10.3%), 1.9% (95% CI: 0.9%–4.1%), and 0.9% (95% CI: 0.4%–2.0%), with heterogeneity (I‐squared [*I* ^2^] = 86.565, *I* ^2^ = 88.460, *I* ^2^ = 73.835, *p* < 0.0001 for all). The evaluated pooled prevalence of NDM, VIM, and IMP was highest in Arak (29.8%; 95% CI: 22.3%–38.5%), Babol (30.0%; 95% CI: 19.0%–44.0%), and Dezful (18.8%; 95% CI: 8.7%–35.9%), respectively.

**Conclusion:**

The increasing prevalence of *NDM* among encoding genes of MBL in *Klebsiella pneumoniae* in Iran represents a major clinical obstacle to therapeutic options in the future. These findings highlight the urgent need for effective infection control measures and the development of new treatment strategies to address the rising resistance. Enhanced surveillance and the implementation of targeted antibiotic stewardship programs are crucial in controlling the spread of these resistant strains. However, the study has limitations, including the potential biases related to publication bias and heterogeneity across studies, which should be considered when interpreting the findings.

## 1 Introduction

The increasing antimicrobial resistance in *Klebsiella pneumoniae* has emerged as a global concern, with the World Health Organization (WHO) designating it as a critical‐priority pathogen due to its rapid spread and limited treatment options [1, 2]. Particularly alarming is the rise in carbapenem resistance, primarily mediated by metallo‐β‐lactamases (MBLs), which complicates treatment options for severe infections [3, 4].

MBLs, including Imipenemase (*IMP*), New Delhi metallo‐β‐lactamase (*NDM*), and Verona integron‐encoded metallo‐β‐lactamase (*VIM*) types, have become significant in the bacterium due to their ability to hydrolyze carbapenems, which are last‐resort antibiotics used to treat severe infections [5]. Multidrug‐resistant strains pose a perilous challenge, requiring comprehensive, innovative strategies for the development of efficient treatment strategies [6].

These antibiotics, part of the β‐lactam group, are structurally related to penicillins but have a broader spectrum of activity against both Gram‐negative and Gram‐positive bacteria [7]. The main characteristics of carbapenems include stability against many β‐lactamases and the ability to penetrate bacterial cell walls efficiently [8].

Resistance to these drugs in the species is acquired through various mechanisms. Enzymatic hydrolysis by carbapenemases is the main mechanism. Carbapenemases are classified into classes A, B, and D [9]. Metallo β‐lactamases (MBLs) belong to the Ambler class B with the ability to degrade them as well as most cephalosporins and penicillins. Although this class is not inhibited by β‐lactamase inhibitors, MBLs are inhibited by metal ion chelators [10].

Diverse groups of MBLs, such as Verona integron‐encoded Metallo β‐lactamase (VIM), New Delhi Metallo β‐lactamase (NDM1), Seoul Imipenemase Metallo β‐Lactamase (SIM), and Sao Paulo Metallo β‐lactamase (SPM) have been recognized in this organism [11].

The growing resistance to these antibiotics in this pathogen, driven by MBL production, poses a major public health threat, as they are last‐resort treatments for infections caused by ESBL‐producing this organism [12]. So far, the most effective carbapenemases, regarding the breakdown of these antibiotics and geographical distribution in Ambler class B, are the *VIM*, *IMP*, and *NDM* [13].

The genes responsible for these enzymes are generally carried by integrons. The ability of MBLs to hydrolyze and resist such antibiotics has raised many concerns regarding the expansion of efficient antibiotics [14].

Reflecting this global concern at a national level, Iran has witnessed a notable rise in resistant forms of the bacterium. However, current reports remain fragmented, highlighting the urgent need for comprehensive national data to understand the scope of the problem [15]. Given the importance of awareness regarding the incidence and prevalence rates of encoding genes for MBLs in this organism to select proper prevention and containment options, this meta‐analysis evaluates the prevalence of key MBL‐encoding genes (NDM, VIM, and IMP) in clinical isolates of the pathogen from clinical samples in Iran, aiming to provide a unified estimate to guide more informed public health strategies and antimicrobial stewardship. While individual studies offer valuable insights, they often present varying results due to differences in methodologies and geographical settings. Therefore, a meta‐analysis is essential to integrate diverse data and provide a more accurate estimate [16].

## 2 Materials and Methods

### 2.1 Literature Search

The current meta‐analysis was carried out to assess the prevalence of *Klebsiella pneumoniae* MBL‐producing genes in Iran from 2010 to 2024. The Preferred Reporting Items for Systematic reviews and Meta‐Analyses (PRISMA) guidelines [17] were used to ensure transparency and reproducibility. The completed PRISMA 2020 checklist is provided as Supporting Information S1: Table S1.

We searched databases such as PubMed, Scopus, Wiley Online Library, ScienceDirect, and Google Scholar to select related papers, and to find additional relevant studies bibliographies of included papers were also reviewed manually. We selected published literature in the English language. Papers were screened by three separate researchers, and eligible full‐text publications were chosen. The keywords used for the English database search include “metallo‐β‐lactamase” OR “MBL” AND *“Klebsiella pneumoniae”* AND “Iran”.

### 2.2 Inclusion and Exclusion Criteria

Eligible Literature for inclusion criteria were: (i) peer‐reviewed original articles, (ii) cross‐sectional studies, (iii) studies reporting the prevalence of encoding genes of MBL‐producing *Klebsiella pneumonia*e from human clinical samples of Iranian hospitals, and (iv) accessible full‐text papers. Papers were excluded based on the following criteria: articles unrelated to the main topic, papers that used nonclinical samples, papers that determined the prevalence of MBL‐producing genes in the MBL *Klebsiella pneumoniae* populations, duplicate publications, editorials, case reports, non‐original papers, letters, papers with meaningless results, non‐English language papers, review articles, case series, and papers reporting the prevalence of MBL in phenotyping methods. Of the 2486 records screened, 2362 were excluded based on the predefined eligibility criteria, including irrelevant topic, non‐clinical samples, non‐original studies, duplicate records, and lack of sufficient or clear data.

### 2.3 Data Extraction

We studied the full texts or abstracts of the selected papers, and the following items were extracted from each paper: author's name, year of publication, source of sample, sample size, detected ESBL genes, and Iranian province.

### 2.4 Quality Assessment and Statistical Analysis

The quality assessment of papers was carried out using the Newcastle‐Ottawa Scale (Table 1) [18]. The scoring process included three sections: (i) selection (maximum of 5 stars), (ii) comparability (maximum of 2 stars), and (iii) outcome (maximum of 3 stars), each with its related score range. The total score for the studies was categorized as follows: 0–4 (*unsatisfactory*), 5–6 (*satisfactory*), 7–8 (*good*), and 9–10 (*very good*) [19].

**Table 1:** Quality assessment using the Newcastle–Ottawa scale modified for cross‐sectional studies.

No.	First author	Year	Selection^a^ (maximum 5 stars)	Comparability^b^ (maximum 2 stars)	Outcome^c^ (maximum 3 stars)	Total score
1	H Zeighami	2014	**	**	***	7
2	H Alizadeh	2021	**	*	**	5
3	S Armin	2018	**	*	**	5
4	S Armin	2021	**	*	**	5
5	E Abbasi	2023	**	*	**	5
6	N Bahmani	2019	**	**	**	6
7	A Bahramian	2019	**	*	**	5
8	N Darabi	2019	***	*	***	7
9	S Davoudabadi	2023	**	**	***	7
10	H Fazeli	2015	**	*	**	5
11	F Firoozeh	2017	**	**	***	7
12	A Hashemi	2014	**	*	**	5
13	Z Hosseinzadeh	2017	**	**	***	7
14	S Jamali	2020	**	**	***	7
15	S Kadivarian	2023	**	*	***	6
16	H Kazemian	2019	**	*	***	6
17	S Kiaei	2018	**	*	**	5
18	B Latifi	2020	**	**	**	6
19	M Moghadampour	2018	**	**	**	6
20	S Nobari	2014	**	*	**	5
21	R Rajabnia	2015	**	*	***	6
22	F Shahcheraghi	2012	**	*	**	5
23	S Shoja	2022	**	*	**	5
24	S Shoja	2017	**	*	***	6
25	F Riyahi Zaniani	2022	***	**	***	8

^a^ Selection (Maximum 5 stars): Representativeness of the sample, Sample size, Non‐respondents, Ascertainment of the exposure (risk factor).

^b^ Comparability (Maximum 2 stars): The subjects in different outcome groups are comparable, based on the study design or analysis. Confounding factors are controlled.

^c^ Outcome (Maximum 3 stars): Assessment of the outcome, Statistical test.

Comprehensive Meta‐Analysis Version 3 (CMA) software was used for statistical analysis. The total number of *Klebsiella pneumoniae* isolates under study was used as the sample size of the selected studies, and the number of genes reported in the studies was used as events.

The heterogeneity among studies was assessed using the *I* ^2^ test, and based on its results, random‐effects or fixed‐effects models were used. In this meta‐analysis, the *I* ^2^ statistic was used to assess heterogeneity, indicating the proportion of variation across studies due to heterogeneity rather than chance. *I* ^2^ values range from 0% (no heterogeneity) to 100% (high heterogeneity). A random‐effects model is applied when *I* ^2^ > 50%, and a fixed‐effects model is used when *I* ^2^ < 50%, ensuring appropriate statistical analysis based on study variability [20].

Egger's regression test and Begg's test were used to evaluate publication bias. Subgroup analysis was performed based on the city. A *p*‐value of less than 0.05 was considered statistically significant, which was two‐sided. The analysis based on the city was conducted as an exploratory analysis and was not pre‐specified in the beginning study design.

## 3 Results

### 3.1 Characteristics of Included Studies

**Figure 1:**
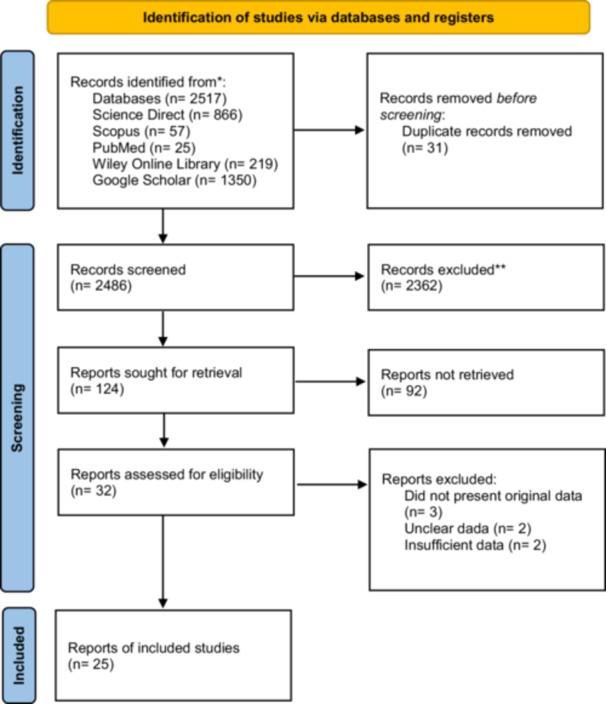
Schematic presentation of article screening.

This study involved a systematic search that identified 2517 papers. Among them, 32 full‐text papers were retrieved for assessment to determine eligibility. After evaluation, two papers were excluded due to a lack of sufficient data, two others were excluded due to unclear data, and three papers did not present original data, such as reviews and workshop reports. Finally, 25 papers [21, 22, 23, 24, 25, 26, 27, 28, 29, 30, 31, 32, 33, 34, 35, 36, 37, 38, 39, 40, 41, 42, 43, 44, 45] met the appraisal criteria for inclusion in the meta‐analysis (Figure 1).

The largest number of reports on the prevalence of encoding genes of MBL‐producing *Klebsiella pneumoniae* in clinical samples in Iran were related to Tehran (five studies), followed by Isfahan (three studies). The studies included in this meta‐analysis employed a molecular approach (conventional PCR and multiplex PCR) (Table 2).

**Table 2:** Main characteristics of the included studies reporting the prevalence of MBL genes in *Klebsiella Pneumoniae*.

No.	Study name	Publication year	Source of sample	Sample size	Genotype methods	MBL genes			Iranian province	
						IMP	VIM	NDM	
1	H Zeighami	2014	Clinical sample	149	PCR Amplification	12	5	NI	Zanjan	
2	H Alizadeh	2021	Clinical sample	240	PCR Amplification	2	11	2	Isfahan	
3	S Armin	2018	Clinical sample	145	PCR Amplification	ND	ND	4	Tabriz/Mashhad	
4	S Armin	2021	Clinical sample	230	PCR Amplification	1	ND	29	Ten Provinces	
5	E Abbasi	2023	Clinical sample	121	PCR Amplification	ND	ND	36	Arak	
6	N Bahmani	2019	Clinical sample	114	PCR Amplification	1	4	NI	Kurdistan	
7	A Bahramian	2019	Clinical sample	120	PCR Amplification	ND	ND	3	Tehran	
8	N Darabi	2019	Clinical sample	182	PCR Amplification	3	22	7	Urmia	
9	S Davoudabadi	2023	Clinical sample	52	PCR Amplification	ND	ND	6	Tehran	
10	H Fazeli	2015	Clinical sample	112	PCR Amplification	ND	ND	6	Isfahan	
11	F Firoozeh	2017	Clinical sample	181	PCR Amplification	NI	NI	20	Kashan	
12	A Hashemi	2014	Clinical sample	83	PCR Amplification	NI	NI	ND	Tehran	
13	Z Hosseinzadeh	2017	Clinical sample	211	PCR Amplification	ND	NI	27	Shiraz	
14	S Jamali	2020	Clinical sample	68	PCR Amplification	NI	NI	8	Guilan	
15	S Kadivarian	2023	Clinical sample	62	PCR Amplification	1	ND	NI	Kermanshah	
16	H Kazemian	2019	Clinical sample	90	PCR Amplification	1	30	ND	Tehran/Ilam	
17	S Kiaei	2018	Clinical sample	175	PCR Amplification	ND	ND	37	Kerman	
18	B Latifi	2020	Clinical sample	151	PCR Amplification	ND	ND	11	Bushehr	
19	M Moghadampour	2018	Clinical sample	80	PCR Amplification	ND	ND	8	Isfahan	
20	S Nobari	2014	Clinical sample	180	PCR Amplification	ND	5	3	Tehran	
21	R Rajabnia	2015	Clinical sample	50	PCR Amplification	NI	15	NI	Babol	
22	F Shahcheraghi	2012	Clinical sample	45	PCR Amplification	NI	NI	1	Tehran	
23	S Shoja	2022	Clinical sample	400	PCR Amplification	ND	ND	30	Bandar Abbas	
24	S Shoja	2017	Clinical sample	170	PCR Amplification	ND	ND	4	Bandar Abbas	
25	F Riyahi Zaniani	2022	Clinical sample	32	PCR Amplification	6	ND	9	Dezful	

Abbreviations: ND, not detected; NI, not investigated.

### 3.2 Prevalence of Encoding Genes of MBL (NDM, VIM, and IMP)

**Figure 2:**
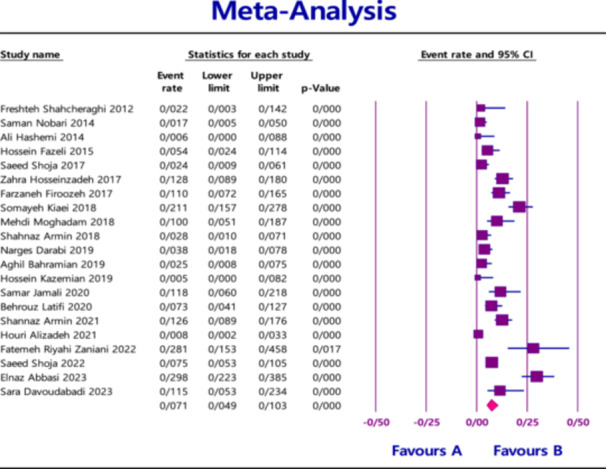
Overall prevalence of *NDM* based on the papers evaluated in the meta‐analysis.

**Figure 3:**
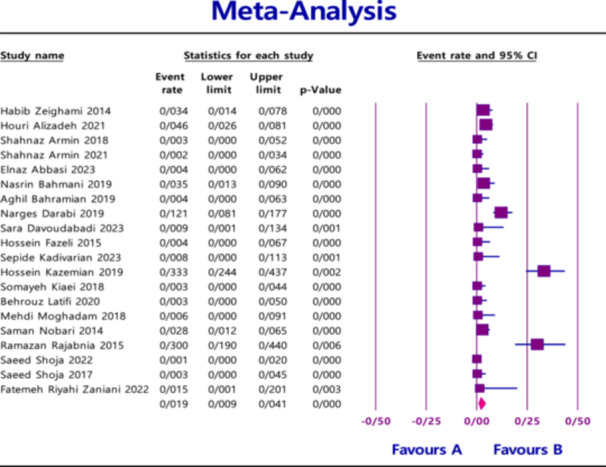
Overall prevalence of *VIM* based on the papers evaluated in the meta‐analysis.

**Figure 4:**
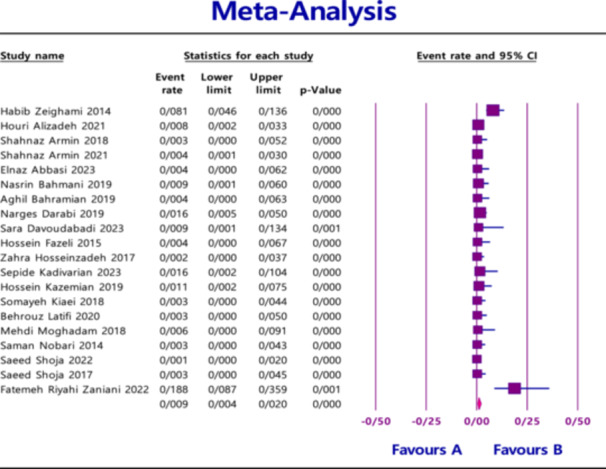
Overall prevalence of *IMP* based on the papers evaluated in the meta‐analysis.

The pooled prevalence of MBL‐producing gene variants in *Klebsiella pneumoniae* in clinical samples was as follows: 7.1% (95% confidence interval [CI] = 4.9%–10.3%) for *NDM* with heterogeneity (*I* ^2^ = 86.565; *p* < 0.0001) (Figure 2), 1.9% (95% CI = 0.9%–4.1%) for *VIM* with heterogeneity (*I* ^2^ = 88.460; *p* < 0.0001) (Figure 3), and 0.9% (95% CI = 0.4%–2.0%) for *IMP* with heterogeneity (*I* ^2^ = 73.835; *p* < 0.0001) (Figure 4).

**Figure 5:**
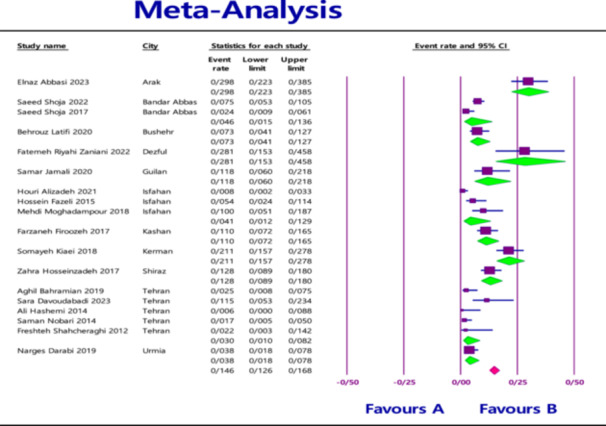
Prevalence of *NDM* based on the city evaluated in the meta‐analysis.

**Figure 6:**
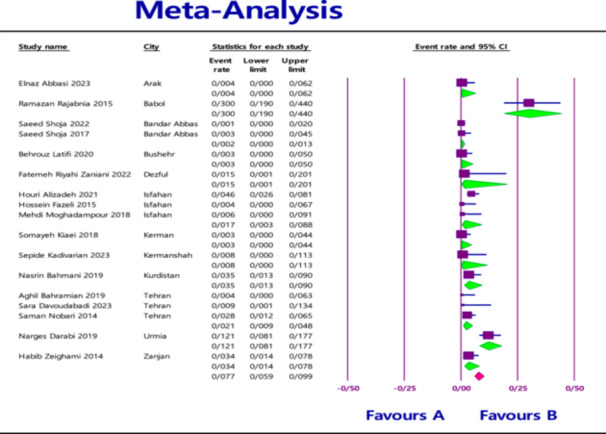
Prevalence of *VIM* based on the city evaluated in the meta‐analysis.

**Figure 7:**
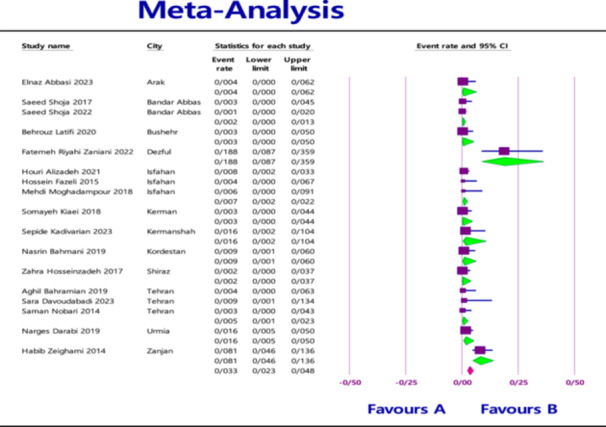
Prevalence of *IMP* based on the city evaluated in the meta‐analysis.

In addition to evaluating the overall prevalence based on genes, another analysis based on the city was performed as an exploratory approach to assess potential regional variations. At the city level the highest prevalence was found in Arak for *NDM* (29.8%; 95% CI = 22.3%–38.5%) with heterogeneity (*I* ^2^ = 87.371; *p* < 0.0001) (Figure 5), in Babol for *VIM* (30.0%; 95% CI = 19.0%–44.0%) with heterogeneity (*I* ^2^ = 83.261; *p* < 0.0001) (Figure 6), and in Dezful for *IMP* (18.8%; 95% CI = 8.7%–35.9%) with heterogeneity (*I* ^2^ = 75.412; *p* < 0.0001) (Figure 7).

### 3.3 Quality Assessment

After assessing the quality of the studies, we found that out of the 25 papers, 18 had an overall score of 5–6 (*satisfactory studies*), and 7 had an overall score of 7–8 (*good studies*) (Table 1). None of the included papers was considered very good or unsatisfactory.

### 3.4 Publication Bias

**Figure 8:**
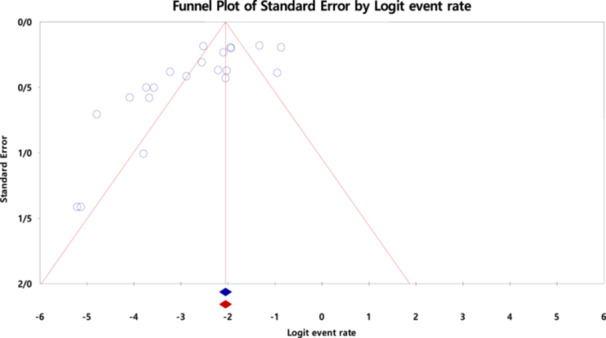
Funnel plot asymmetry based on the most prevalent gene of MBL (*NDM*).

**Figure 9:**
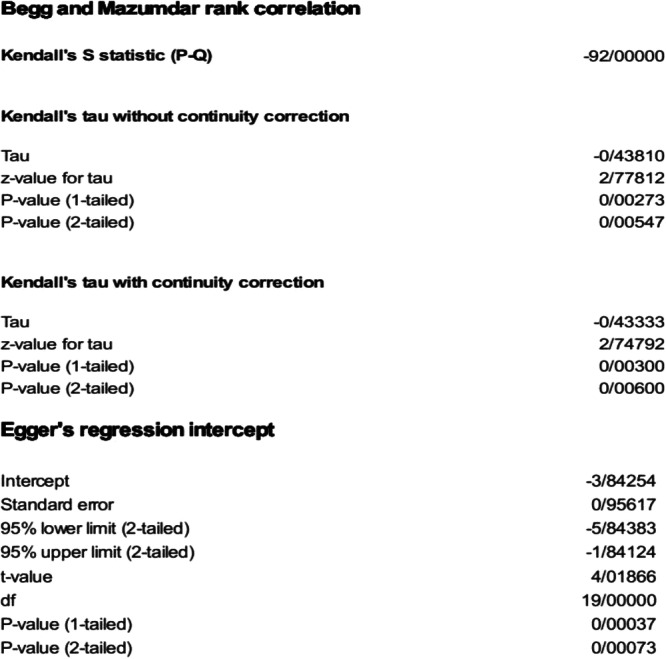
Begg and Mazumdar rank correlation and Egger regression intercept analysis for the prevalence of *NDM*.

**Figure 10:**
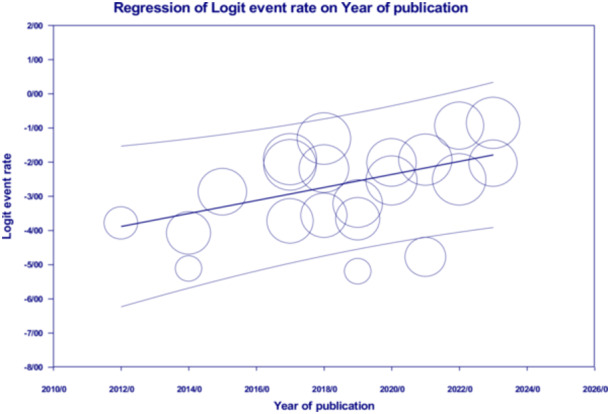
A meta‐regression graph for the prevalence of *NDM* based on the year of publication.

In this study, the funnel plot (Figure 8) asymmetry was assessed for the most prevalent gene of MBL (*NDM*) using Begg's (*p* = 0.006) and Egger's tests (*p* = 0.0007), indicating evidence of publication bias (Figure 9). There was variability between studies in terms of publication year in the meta‐regression analysis for the most prevalent MBL gene (*NDM*) (*p* = 0.009) (Figure 10).

### 3.5 Time Series Analysis

**Figure 11:**
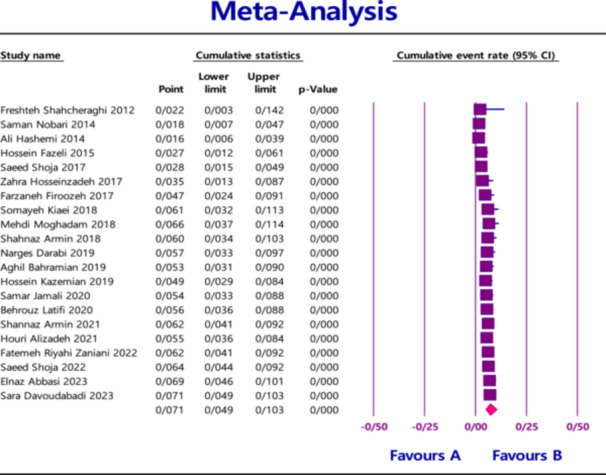
Forest plots for the prevalence of *NDM* during the time.

The trend in the prevalence of *NDM* in *Klebsiella pneumoniae* slightly decreased from 2012 to 2014. This was followed by a noticeable increase between 2015 and 2018. Then, from 2018 to 2019, a slight decline was observed. Finally, from 2019 to 2023, the prevalence increased slightly again (Figure 11).

## 4 Discussion

Modern and recent medicine is threatened by increasing antibiotic resistance even on a global scale, particularly among Gram‐negative bacteria. MBL enzymes are a special concern and are spreading rapidly worldwide. MBL‐producing bacteria have multiple additional drug resistances, leaving few apparent treatment options. Due to the unique structure, function, and diversity of MBLs, MBLs are considered a special challenge for drug development [46].

The findings of this study revealed that *NDM*, with a prevalence of 7.1%, is the most prevalent gene among other encoding genes of MBL‐producing *Klebsiella pneumoniae* in Iran. The observed regional differences in the prevalence of MBL‐encoding genes, particularly NDM, may be attributed to factors such as inappropriate antibiotic use, varying healthcare infrastructure, and geographic characteristics, which warrant further investigation [47].

To better understand the global context, it is important to compare the prevalence of MBL‐producing *Klebsiella pneumoniae* in Iran with data from other regions. The pooled prevalence of NDM in Iran, at 7.1%, is notable but lower than in some high‐prevalence regions like India and Pakistan, where NDM prevalence has been reported to exceed 40% [48]. Conversely, the prevalence of VIM (1.9%) in Iran is lower compared to several European countries, including Poland and Greece, where rates exceeding 20% and 17%, respectively, have been observed [49, 50]. Furthermore, the prevalence of IMP (0.9%) in Iran is among the lowest when compared to certain regions in Asia, such as China, where IMP‐producing strains have been more prevalent [51]. These comparisons underscore the need for focused surveillance and tailored healthcare responses to address the growing threat of antibiotic resistance [52].

The highest and lowest pooled prevalence of NDM‐producing *Klebsiella pneumoniae* was observed in Arak with 29.8% and Tehran with 3.0%, respectively, however, the highest prevalence was based on only one study.

The emergence of *NDM*‐harboring *Klebsiella pneumoniae* in Iran is concerning since such *Klebsiella pneumoniae* displays resistance to antibiotics generally used for treating Gram‐negative infections (β‐lactams, aminoglycosides, and fluoroquinolones) and has shown a tendency to spread. Among the encoding genes of MBL, *NDM*‐producing *Klebsiella pneumoniae* is regarded as the most predominant gene [53]. Recent studies reporting *NDM* as the most prevalent carbapenemases among carbapenem‐resistant *Klebsiella pneumoniae* isolates in Iran [25, 54], which is in line with our results.

The increasing antibiotic resistance in *Klebsiella pneumoniae* poses a significant threat to public health, as these multidrug‐resistant strains limit treatment options and exacerbate the difficulty in managing infections, especially in hospital settings [55].

In our study, out of 20 studies that investigated *VIM*, the presence of *VIM* was identified in only 5 studies. In a study conducted by Rajabnia, the prevalence of VIM‐producing carbapenemases among *Klebsiella pneumoniae* isolates from intensive care unit patients was found to be 30% (15/50 samples) [40], which is much higher than reported in several other parts of Iran.

Although the pooled prevalence of VIM‐producing *Klebsiella pneumoniae* in our study was notably less than *NDM*, it is important to highlight that VIM‐producing *Klebsiella pneumoniae* has a high potential to spread among intensive care unit patients with severe underlying conditions [56], which was confirmed in the Rajabnia study. Over the last decade, VIM‐type MBLs have diffused in *Klebsiella pneumoniae* [57, 58], and outbreaks have been reported [59].

When compared to other global studies, our findings align with the increasing prevalence of NDM‐producing *Klebsiella pneumoniae* in many regions, highlighting the critical need for enhanced surveillance and intervention strategies in Iran [25, 36]. IMP‐producing *Klebsiella pneumoniae* has been recognized worldwide and has caused some small‐scale outbreaks [60, 61, 62]. The prevalence of IMP‐producing *Klebsiella pneumoniae* varies by location and primarily causes outbreaks in clinical settings [63].

Our meta‐analysis showed that, among the 20 papers included in this study, only 8 papers reported IMP‐type MBLs, with the lowest prevalence of *IMP* at 0.9%. This suggests that IMP‐producing *Klebsiella pneumoniae* exhibits a sporadic prevalence in Iran. In a study conducted by Lai et al. [64], only 0.19% (25/537) of isolated *Klebsiella pneumoniae* strains produced *IMP*, which is in line with our results.

Effective surveillance and antimicrobial stewardship strategies are crucial to controlling the spread of resistant strains, particularly NDM‐producing *Klebsiella pneumoniae*, to mitigate the risks associated with the limited treatment options [65].

The results of this meta‐analysis should be interpreted with caution, considering certain limitations. First, the observed publication bias likely stemmed from either the lack or the limited number of papers available from Iranian cities. The second limitation is that our study focused exclusively on English‐language publications. The third is that some of the studies included in our analysis exhibited small‐study effects, which may be related to a limited sample size.

Despite these limitations, it is important to acknowledge that this study provides the most comprehensive insights into the prevalence of MBL‐encoding genes produced by *Klebsiella pneumoniae* in clinical samples in Iran. Moreover, differences in diagnostic methods (e.g., PCR protocols, sample sources) and potential overrepresentation of certain hospitals or regions may have influenced the pooled results [66]. These factors should be considered when interpreting the findings.

Future studies should focus on the identification of underlying factors driving the spread of MBL‐encoding genes in *Klebsiella pneumoniae*, as well as the development of alternative treatment strategies to counteract these resistant strains [67].

In conclusion, the high prevalence of NDM indicates that, in the absence of efficient therapy, surveillance gains added value in controlling and preventing the spread of NDM‐producing isolates with increased pathogenic profiles. Hospitals, public health agencies, and healthcare professionals should take the lead in implementing enhanced surveillance and intervention measures to effectively monitor, report, and control the spread of these resistant strains.

## Author Contributions

**Hamid Sadeghi:** data curation, formal analysis, investigation, methodology, writing – original draft, software. **Susan Khanjani:** investigation, methodology, writing – original draft. **Masoumeh Aslanimehr:** investigation, methodology, validation. **Nima Aria:** writing – original draft, investigation, methodology. **Mohammad Reza Maleki:** investigation, methodology. **Farhad Nikkhahi:** methodology, investigation. **Nematollah Gheibi:** methodology, investigation. **Seyed Mahmoud Amin Marashi:** investigation, methodology. **Saeideh Gholamzadeh khoei:** conceptualization, writing – review & editing, funding acquisition, project administration, supervision.

## Conflicts of Interest

The authors declare no conflicts of interest.

## Transparency Statement

The lead author Saeideh Gholamzadeh khoei affirms that this manuscript is an honest, accurate, and transparent account of the study being reported; that no important aspects of the study have been omitted; and that any discrepancies from the study as planned (and, if relevant, registered) have been explained.

## Supporting information

Supplementary Table 1.

## Data Availability

The data that support the findings of this study are available from the corresponding author upon reasonable request.
